# Dynamic 3D Locus Organization and Its Drivers Underpin Immunoglobulin Recombination

**DOI:** 10.3389/fimmu.2020.633705

**Published:** 2021-02-18

**Authors:** Carolyn H. Rogers, Olga Mielczarek, Anne E. Corcoran

**Affiliations:** ^1^ Lymphocyte Signalling and Development Programme, Babraham Institute, Cambridge, United Kingdom; ^2^ Nuclear Dynamics Programme, Babraham Institute, Cambridge, United Kingdom

**Keywords:** V(D)J recombination, immunoglobulin, 3D conformation, loop extrusion, RAG scanning, chromatin regulation, genome organization

## Abstract

A functional adaptive immune system must generate enormously diverse antigen receptor (AgR) repertoires from a limited number of AgR genes, using a common mechanism, V(D)J recombination. The AgR loci are among the largest in the genome, and individual genes must overcome huge spatial and temporal challenges to co-localize with optimum variability. Our understanding of the complex mechanisms involved has increased enormously, due in part to new technologies for high resolution mapping of AgR structure and dynamic movement, underpinning mechanisms, and resulting repertoires. This review will examine these advances using the paradigm of the mouse immunoglobulin heavy chain (Igh) locus. We will discuss the key regulatory elements implicated in Igh locus structure. Recent next generation repertoire sequencing methods have shown that local chromatin state at V genes contribute to recombination efficiency. Next on the multidimensional scale, we will describe imaging studies that provided the first picture of the large-scale dynamic looping and contraction the Igh locus undergoes during recombination. We will discuss chromosome conformation capture (3C)-based technologies that have provided higher resolution pictures of Igh locus structure, including the different models that have evolved. We will consider the key transcription factors (PAX5, YY1, E2A, Ikaros), and architectural factors, CTCF and cohesin, that regulate these processes. Lastly, we will discuss a plethora of recent exciting mechanistic findings. These include Rag recombinase scanning for convergent RSS sequences within DNA loops; identification of Igh loop extrusion, and its putative role in Rag scanning; the roles of CTCF, cohesin and cohesin loading factor, WAPL therein; a new phase separation model for Igh locus compartmentalization. We will draw these together and conclude with some horizon-scanning and unresolved questions.

## V(D)J Recombination and the Igh Locus

V(D)J recombination is catalyzed by the recombinase complex, comprising recombination-activating genes 1 and 2 (RAG1-RAG2), that cleaves recombination signal sequences (RSSs) flanking antigen receptor (AgR) genes (1). Referred to as the 12/23 rule, recombination normally occurs exclusively between a 12-RSS and a 23-RSS, thereby for example enabling recombination of Igh D_H_ genes, flanked on both sides by 12-RSS, with both J_H_ and V_H_ genes, flanked by 23-RSS, and preventing direct recombination between V_H_ and J_H_ genes with identical RSSs. Recombination is focused at recombination centers: regions of highly concentrated RAG binding which orchestrate RSS synapsis (2). V(D)J recombination is tightly regulated: it is lineage- and cell stage-specific and is controlled at three distinct levels. The first is the developmental stage-specific and cell cycle-regulated expression of the RAG recombinase (3–5). Secondly, next generation repertoire sequencing methods have revealed that several mechanisms including transcription factor binding, ATP-dependent chromatin remodeling, histone modifications, DNA methylation and non-coding transcription, regulate the local accessibility of AgR gene RSSs to the RAG recombinase within chromatin (6–8). However changes in local chromatin accessibility alone are insufficient to promote V(D)J recombination of spatially separated V, D and J genes (9). The third level of regulation is the spatial genomic organization of the AgR loci. Understanding the architecture of the AgR loci is an enormous technical challenge: they are large, multimegabase, repetitive loci that, given their biological function, are expected to exhibit multiple conformations. The dynamic 3D conformation of the Igh locus and its contributory mechanisms will be the main focus of this review, in part because studies of AgR conformation have made most progress in this locus, but given the countless similarities among them, the principles discussed here will be a very relevant paradigm for the other AgR loci.

**Figure 1:**
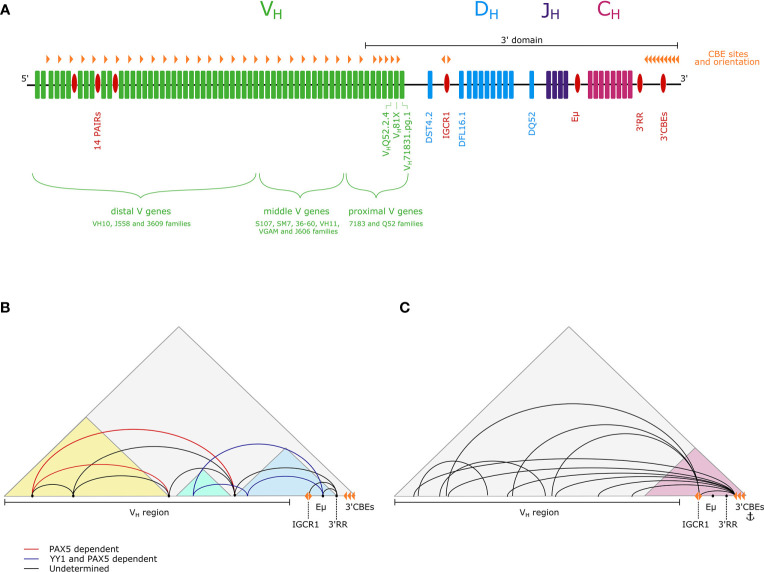
**(A)** A schematic of the Igh locus showing V_H_ (green), D_H_ (blue), J_H_ (purple) and C_H_ (pink) genes, as well as regulatory elements (red). The positions of CBEs are also marked (orange triangles).The 5’-3’ orientation is indicated and the extent of the 3’domain is depicted by a black line. **(B)** The three subdomain model proposes that within the Igh TAD (gray) there are three subdomains (light blue, light green, yellow), with extensive looping within them. Interactions between subdomains are mediated by loop anchoring sites (black points). A subset of these loops is PAX5-dependent (red) and PAX5- and YY1-dependent (dark blue) (10–12). **(C)** The flexible looping model proposes that there is a dynamic continuum of loops from the entire V_H_ region to the 3’ end of the locus, with the 3’ region forming a more structurally invariant subdomain (pink) containing an anchor point for long-range interactions (13, 14).

The mouse immunoglobulin heavy chain (Igh) locus comprises 195 variable V_H_ genes in 16 families, 10 diversity D_H_ genes, 4 joining J_H_ genes and 8 constant C_H_ genes (**Figure 1A**) (15). To generate antibody diversity, the V_H_, D_H_, and J_H_ genes undergo V(D)J recombination in a strictly regulated, sequential manner. First, D_H_-J_H_ recombination commences in early pre-pro-B lymphoid progenitors and is completed by the early pro-B cell stage on both Igh alleles. Subsequently, one Igh allele undergoes V_H_-DJ_H_ recombination in committed pro-B cells. The second allele undergoes allelic exclusion. A successful recombination event is achieved when the newly produced heavy chain polypeptide pairs with a surrogate light chain and is expressed as a pre-B cell receptor (pre-BCR) on the cell surface. If this first V_H_-DJ_H_ recombination event is unsuccessful, then recombination is attempted on the remaining allele. Thereafter the Igκ locus (3Mb; 140 V_κ_ genes, 5 J_κ_ genes) undergoes a single recombination event—V_κ_-J_κ_—in pre-B cells to generate an Igκ polypeptide. Pairing of Igh and Igκ generates the BCR (16).

There are a number of well characterized regulatory elements in the Igh locus that contribute to locus structure (**Figure 1A**). First, the intronic enhancer Eμ, located between the J_H_ and C_H_ genes (17), is required for effective recombination (18–21). Second, the 3’ regulatory region (3’RR), which contains four DNase I hypersensitive sites (HSs), HS1-4, is a B lineage-specific enhancer (22, 23). Downstream, there is a regulatory element, containing HS5-8 and 10 binding sites for CCCTC-binding factor (CTCF), which is known as the 3’ CTCF binding elements (3’CBEs) and functions as an insulator and a superanchor (13, 24). In addition, the intergenic control region 1 (IGCR1), located just upstream of the D_H_ genes, contains two CBEs, and operates as an insulator to prevent premature V_H_-DJ_H_ recombination (25). Potential regulatory elements have also been discovered in the distal V_H_ region, in the form of 14 tandemly repeating PAX5-activated intergenic repeat (PAIR) elements (26). PAIR elements are bound by several transcription factors and architectural proteins, including the B cell-specific transcription factor PAX5, which mediates non-coding antisense transcription from PAIR elements in pro-B cells (26, 27).

## Early Fluorescent In Situ Hybridization (FISH) Experiments Revealed Subnuclear Relocation, Locus Contraction, and DNA Looping in the Igh Locus

Local chromatin environments within the nucleus can be permissive for or refractory to active nuclear processes. The nuclear periphery is a region of repressive heterochromatin, and in non-B cells the Igh locus is anchored at the nuclear periphery *via* its V_H_ end (28). FISH studies showed that before V(D)J recombination, the Igh locus is activated by relocation from the nuclear periphery to permissive euchromatin in the nuclear interior (29, 30), dependent on the transcription factors EBF1 and E2A, and on Eμ (10, 29, 31–33). In addition to relocation, the Igh locus undergoes large-scale contraction to bring V_H_ and D_H_ genes in spatial proximity for V_H_ to DJ_H_ recombination (29, 30, 34). This was measured by a significant reduction in the distance between FISH probes positioned on opposite ends of the locus in pro-B cells compared with pre-pro-B cells. DNA looping was subsequently visualized with three-color FISH, which showed that distal V_H_ genes frequently loop closer to the C_H_ region than proximal V_H_ genes that are closer in linear sequence (33). These early FISH studies introduced pro-B cells lacking the RAG recombinase as a model to study Igh locus interactions. In *Rag* ^-/-^ pro-B cells, the Igh locus retains its intact germline sequence but is poised for recombination.

Translating individual observations of Igh locus contraction and looping into a comprehensive model of the 3D structure of this enormous locus has been a challenge. The Murre laboratory first visualized a putative overall 3D Igh locus structure using 11 small FISH probes distributed evenly along the Igh locus, combined with mathematical modeling (35). Distal V_H_ probes were more frequently positioned close to the Eμ-J_H_ region in pro-B cells than in pre-pro-B cells, revealing a major conformational change in the Igh locus when it is poised for recombination, suggesting that the distal V_H_ genes are brought close to Eμ-J_H_ region to have equal opportunity with proximal V_H_ genes to recombine. Furthermore, in pro-B cells, the 3’ end of the Igh locus interacted with distal V_H_ genes as frequently as with the proximal V_H_ genes, indicating extensive long-range DNA looping involving this CBE-rich region. This study also reported larger variability of distance measurements in pro-B cells than in pre-pro-B cells, suggesting more variation in the interaction landscape at this stage. Moreover, the locus formed into clusters of loops at both developmental stages.

Subsequently, the Murre lab employed live imaging of fluorescently labeled Igh loci and computer modeling to determine first passage times of interactions between V_H_ and D_H_ genes (36). This is a major advance beyond FISH techniques on fixed cells, giving insights into the real-time dynamics of recombination. This study revealed that a V_H_ gene interacts with the DJ_H_ region within minutes of D_H_-J_H_ recombination, much faster than expected. It was proposed that the D_H_J_H_ region resides in a cavity surrounded by equally distant V_H_ genes and that the viscous nuclear environment causes V_H_ genes to bounce back and forth rapidly until a specific synapsis is established by the RAG machinery, aligning with the equal opportunity model above (37).

## DNA Looping Mediators

Several mouse models have given insight into the transcription factors required for locus contraction and looping. PAX5, YY1, E2A and Ikaros are essential for contraction and all bind to numerous sites within the Igh locus, but it remains unclear if their roles are direct or indirect (33, 34, 38, 39). CTCF is also implicated in mediating loops in the Igh locus: reduction of CTCF binding decreases locus contraction (40). CTCF binding to the Igh locus is lymphocyte-specific (41). The 10 CBEs in the 3’CBEs (13, 41) are in a convergent orientation to 121 CBEs distributed throughout the V_H_ region (41), likely facilitating looping between the 3’ and 5’ ends of the locus. In addition, there are two CBEs in the IGCR1 that are in divergent orientations: CBE1 can facilitate CTCF-mediated looping between the IGCR1 and the V_H_ region, while CBE2 can facilitate interactions between IGCR1 and the 3’CBEs (13, 14, 42). There is extensive co-localization between CTCF and cohesin binding sites genome-wide (43), and cohesin binds to multiple shared sites within the Igh locus, although its binding is more dynamic, being most enriched at the pro-B stage (44).

## 3C Based Technologies Provide Higher Resolution Information on These Loops

Detailed understanding of the 3D DNA interactions governing AgR recombination have benefited enormously from chromosome conformation capture (3C) and its later refinements (4C, 5C, HiC), which have revealed genomic contacts underpinning locus contraction at higher resolution (10s of kb compared with 100s of kb for FISH).

3C-based studies have shown that the Igh locus forms a discrete topologically associated domain (TAD) (11, 13). Furthermore, the 3’ domain of the Igh locus is compacted and highly structured, suggesting little variability between individual B cells. The 3’CBEs, 3’RR and IGCR1 interact with Eμ, in addition to a separate interaction between the 3’ CBEs and IGCR1 (10, 40). These interactions are independent of locus contraction (14). However, more recent 4C and 5C studies of Igh locus conformation suggest that the 3’ domain extends beyond the V_H_-D_H_ intergenic region, to encompass proximal V_H_ gene segments (11, 14). This is consistent with the observation that proximal V_H_ genes can undergo recombination in the absence of locus contraction in pro-B cells (34, 45).

There are also local interactions within the V_H_ region. A number of these interactions are independent of locus contraction, such as the PAX5-independent interactions of the PAIR elements with other regions in the distal V_H_ (14). Moreover, middle VH36-60 and distal VH10 regions of the locus are involved in local interactions that are CTCF mediated and are present in Eµ deficient cells with impaired contraction (10, 12).

In addition to local looping, there is a requirement for long range contacts between the 5’ and 3’ ends of the locus to facilitate V_H_-DJ_H_ recombination. How V_H_ genes loop to the 3’ region at the pro-B cell stage to enable all V_H_ genes to recombine is an ongoing debate. A 5C and FISH study from the Kenter lab defined loop anchor sites that are key in mediating megabase-scale interactions between subdomains, and demonstrated that these long-range interactions are Eμ independent, with a subset being PAX5-dependent. On this basis they have proposed the three subdomain model, also favored by the Sen lab (11, 12). They propose compaction is achieved by CTCF-dependent intra-subdomain looping, together with long-range looping between these subdomains, mediated by PAX5 and YY1 (**Figure 1B**) (11, 12, 46).

In contrast, based on observations of interactions from 16 4C-seq viewpoints in the Igh locus, the Busslinger laboratory propose a flexible looping model, with a continuum of dynamic loops from V_H_ gene segments to the more rigid 3’ domain, providing equal opportunity for recombination of all V_H_ genes, aligning with findings from the Murre lab (**Figure 1C**) (14). The Busslinger study also questioned the importance of Eµ and the IGCR1 as central hubs of intralocus interactions.

## Recent Insights Into the Mechanism of Loop Formation in the Igh Locus: RAG Scanning and Loop Extrusion

Building on FISH and 3C studies that have given a detailed understanding of overall Igh locus conformation and intralocus looping events, recent work has investigated the mechanism of loop formation in the locus. In studies aiming to understand RAG off-target activity in chromosomal translocations, the Alt lab showed that RAG off-target activity is limited to convergent CBE-based loop domains (47). 12-RSS and 23-RSS pairs were inserted at random into the genome of a pro-B cell line to create ectopic recombination centers. RAG mediated recombination occurred within convergent CBE loop domains. Furthermore, deletional recombination of RSSs conforming to the 12/23 rule, and in a convergent orientation was preferred, similar to canonical Igh V(D)J recombination. This indicated that RAG may bind one RSS and then linearly track along chromatin to find a convergent RSS within the same loop domain (47). This model is referred to as the RAG scanning hypothesis (48).

A wealth of evidence demonstrates that TADs and finer-scale loop features are formed through the process of loop extrusion (49–51). Cohesin, a loop extruding complex that holds two strands of DNA together, is loaded onto DNA by Nipbl. Cohesin progressively extrudes larger DNA loops until stalled at boundary elements, principally CTCF bound at convergent CBEs. The enrichment for CBEs at key regulatory regions in the Igh locus suggests a role for CTCF loop domains in organizing locus architecture, and the restriction of off-target RAG scanning to loop domains suggests that scanning could also occur through a loop extrusion related process in the Igh locus (47).

**Figure 2:**
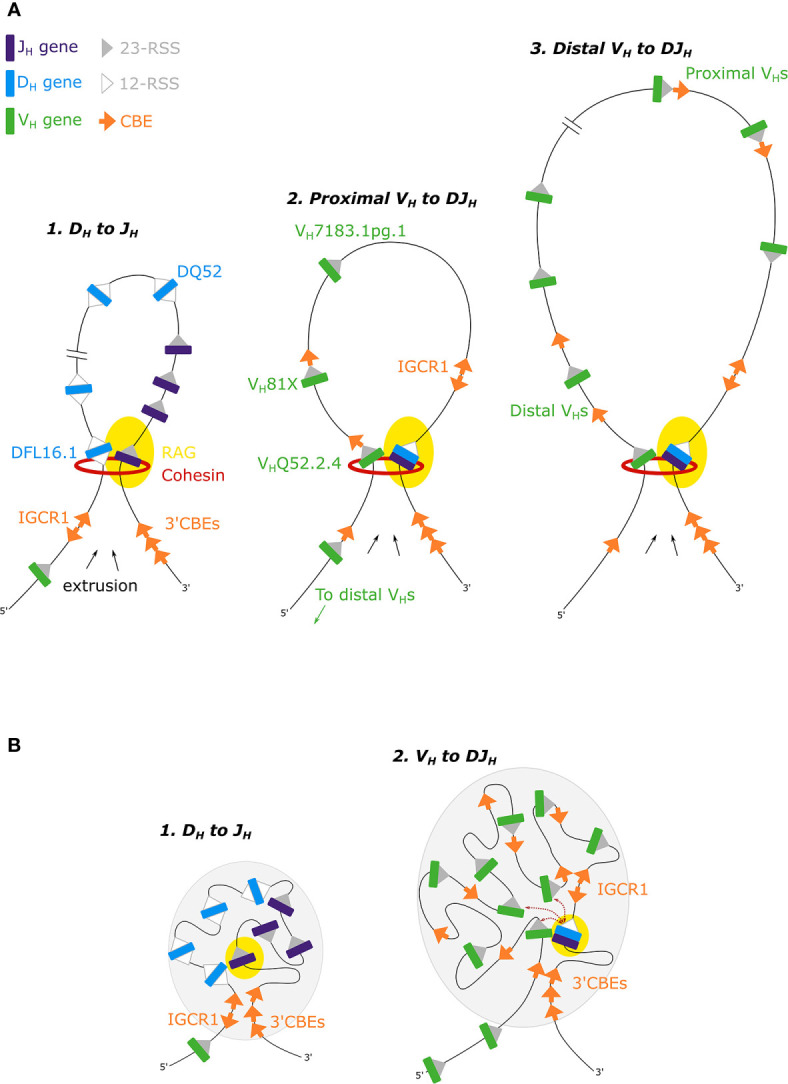
**(A)** The loop extrusion model for D_H_-J_H_, proximal V_H_-DJ_H_ and distal V_H_-DJ_H_ recombination. Cohesin-mediated RAG-scanning leads to the extrusion of progressively larger loops, until loops are stalled by convergent CBEs (53, 54). **(B)** In the phase separation model for Igh recombination, recombination occurs near the boundary of the gel phase (gray ovals) (55).

Mutational analysis showed that inversion of D_H_-12-RSSs impaired D_H_-J_H_ rearrangement, indicating that the orientation of the J_H_-23-RSS directs upstream RAG scanning to a convergently orientated D_H_-12-RSS in the Igh 3’ subdomain (52). Hence in most D_H_-J_H_ rearrangements, recombination by deletion is favored over recombination by inversion, suggesting a role for RAG scanning by loop extrusion (**Figure 2A**). However, recombination from the DQ52 gene segment, adjacent to the J_H_ genes (**Figure 1A**), can be inversional, using a diffusion-based mechanism due to its close spatial proximity to the DJ_H_ recombination center.

A number of recent studies from the Alt and Sen labs have shown that deletion of the IGCR1 leads to increased interactions between the 3’ domain, specifically Eµ, and proximal V_H_ genes, resulting in increased recombination of these proximal genes (56–58). IGCR1 deletion disrupts CBE loop domains within the Igh locus, consequently altering RAG targets (47). Deletion leads to premature V_H_ to germline D_H_ joins, and increased rearrangement of the remote, rarely utilized, D_H_ gene DST4.2 upstream of the IGCR1 (**Figure 1A**). Thus removing this barrier allows extrusion into the intergenic and proximal V_H_ region (58). The Sen lab has also shown that recombination of V_H_ gene segments to germline D_H_ gene segments can occur by inversion of the D_H_ gene, rather than deletion, on IGCR1-deficient alleles (58). They argue that the mechanism of V_H_-DJ_H_ recombination is distinct from that of D_H_-J_H_ recombination, and propose that V_H_-DJ_H_ recombination occurs primarily through a diffusion-based mechanism. However, V_H_-DJ_H_ recombination *via* diffusion has not been shown on wild type alleles, and it is unclear what can be extrapolated from the IGCR1 deletion context.

Other barriers to RAG scanning include active transcription (59), which has been proposed to focus RAG to its target RSSs (52). Transcription impedes RAG scanning in an artificial construct with cryptic scanning across the downstream C_H_ region (52). It will be important to determine whether transcription impedes RAG scanning in canonical locations, for example at the PAIR elements, since this would implicate two bona fide, complementary mechanisms in enabling RAG accumulation at target RSSs.

CTCF binding sites within the V_H_ region exhibit a bipartite pattern. Proximal recombining V_H_ genes are associated with a CBE just 3’ of their RSS but pseudogenes in this region lack adjacent CBEs (37, 44). In contrast, distal V_H_ genes are not closely associated with CBEs, which can either be far upstream of V_H_ genes or intergenic (44, 60). Focusing on the proximal V_H_ genes: mutation of the CBE associated with the first functional V_H_81X gene (**Figure 1A**), leads to reduced V_H_81X gene usage but increased usage of the next upstream gene V_H_Q52.2.4, alongside a reduced interaction between V_H_81X and both the IGCR1 and the 3’CBEs (56, 57). Recombination of the V_H_Q52.2.4 gene is also dependent on its flanking CBE (56). Moreover, repair of a non-functional CBE associated with the first V_H_ gene, pseudogene V_H_7183.1pg.1, converted it into the most highly rearranging V_H_ gene (56). This work supports a RAG scanning mechanism within the proximal V_H_ genes (**Figure 2A**), where convergent CBEs impede further scanning, thereby tethering the RAG complex and mediating interactions between V_H_ genes and the 3’ domain to facilitate V_H_ gene recombination.

Two recent studies have extended our knowledge of RAG scanning within the V_H_ domain, suggesting that it is not limited to the proximal V_H_ genes but occurs over the entire multi-megabase V_H_ region. Both the Busslinger and Alt labs generated large inversions in the V_H_ domain of primary pro-B cells. In the first, there was an 890kb inversion in the distal V_H_ region, while in the second, nearly the entire 2.4Mb murine Igh V_H_ region was inverted (53, 61). In both cases, recombination of the inverted V_H_ genes was abrogated. Efficient recombination was dependent on both the orientation of the V_H_ gene and of the associated CBEs (53), and utilization of cryptic RSSs, now in a convergent orientation, was activated (61). Busslinger and colleagues also used 3C-seq to demonstrate that following inversion of the distal domain, interactions across the entire Igh locus were altered (53). Furthermore, insertion of an array of 20 CBEs in the middle of the Igh V_H_ domain in an inverted orientation altered loop domains in the Igh locus and disrupted long range interactions from the 3’ of the locus to the V_H_ domain. These findings implicate a loop extrusion mechanism for recombination across the entire V_H_ domain (**Figure 2A**), with loops of up to 2.8Mb (53), larger than the average TAD (880kb) (62). However, while recombination to V_H_ genes immediately 5’ of the insertion was reduced, this was limited to a 355kb region, which is not consistent with a single looping event across the V_H_ domain (53). In addition, FISH and 4C studies indicate multiple loops in the distal V_H_ domain (14, 35). It remains unclear how large numbers of CBEs are bypassed to generate loops between distal V_H_ genes and the DJ_H_ gene segment. It is also unknown how frequently the >100 CBEs in the V_H_ region are occupied by CTCF. If occupation follows a dynamic pattern on individual Igh alleles, this would facilitate a stochastic pattern of CBE stalling that would enable diverse participation of V_H_ genes in V_H_-D_H_ recombination. It also remains unclear how loop extrusion across the entire V_H_ domain enables the recombination of all active distal V_H_ genes given that they are not paired with a CBE. Does loop extrusion work in concert with diffusion based mechanisms for distal V_H_ genes, whereby stalling of the loop acts to tether a number of genes in close proximity to the recombination center, obviating the need for juxtaposed CBEs? It is also feasible that other loop impeding mechanisms, such as transcription (59), may contribute to stalling. All of these possibilities require experimental testing.

Clarification of the roles of CTCF and cohesin in RAG scanning by loop extrusion has come from degradation of Rad21 and CTCF in v-Abl-transformed pro-B cell lines, using an auxin-inducible degron system (54). These cell lines have impaired locus contraction and favor proximal over distal V_H_-DJ_H_ recombination compared to primary pro-B cells, despite the distal V_H_ genes being highly transcribed; the underlying defect is not known (54). Rad21 degradation led to: disruption of looping in the 3’ region; severe reduction in D_H_-J_H_ recombination (DQ52 could still recombine through diffusion based mechanisms); and total abrogation of V_H_-DJ_H_ recombination. This supports a role for cohesin-mediated loop extrusion in both stages of V(D)J recombination, albeit in a system where only the most proximal five V_H_ genes recombine. CTCF degradation reduced both CTCF and Rad21 occupancy in the V_H_ region (54). Strikingly, while CTCF mediated interactions were reduced, interactions of the recombination center with V_H_ regions were increased. Distal V_H_-DJ_H_ recombination was restored, comparable to wild type loci in primary pro-B cells that undergo extensive locus contraction. Bound CTCF impedes RAG scanning at the 3’ V_H_ genes (56), so relief of this impediment through CTCF degradation may promote cohesin-mediated RAG scanning throughout the V_H_ region. However this interesting study raises some puzzling questions. How does restoration of loop extrusion and distal V_H_ gene recombination caused by loss of CTCF binding in a v-Abl-transformed pro-B cell line equate with the wild type *in vivo* situation where CTCF is bound and recombination to distal V_H_ genes occurs? Loss of CTCF also caused global reorganization of chromatin, thus it cannot be ruled out that the effect of CTCF loss at the Igh locus in the cell lines is an indirect effect of expression of another factor, or of more widespread genome disorganization. CTCF binds the Igh locus earlier in development with cohesin binding more dynamically (41). Moreover, in order to fully understand how diverse antibody repertoires are generated, where loop stalling may be a stochastic process across a population of cells, the occupancy of CTCF and cohesin at a single cell or allele level, the interplay between these two factors, which bind similar motifs, and their binding kinetics need further investigation. Furthermore, in contrast, while beyond the scope of this review, loss of CTCF *in vivo* leads to impaired recombination of distal Vκ genes (63). Taken together, while it is clear that CTCF plays a pivotal role in organizing Igh (and Igκ) locus structure, how this is achieved requires further investigation.

## New Roles for Transcription Factors in Genome Organization Impact Igh Conformation

Reframing the model from a direct to an indirect role has recently occurred for PAX5, a key transcription factor that mediates commitment to the pro-B cell stage, and long implicated in control of the 3D organization of the Igh locus (34). As PAX5 binds to multiple sites in the Igh locus, including the 3’RR, the V_H_-D_H_ intergenic region (but not at the IGCR1) and the distal V_H_ region at the PAIR elements and at approximately 20 V_H_ gene RSSs (6, 26), it has been hypothesized that its importance in Igh recombination is due to direct regulation of the locus (26, 27). However recent insights from the Busslinger lab indicate that PAX5 regulates Igh recombination, at least in part, indirectly through control of the cohesin DNA release factor WAPL (53). PAX5 represses the transcription of Wapl in both pro- and pre-B cells, through binding to the Wapl promoter and recruiting PRC2. PAX5 downregulation of Wapl increases cohesin residence time, and modulates Igh loop extrusion and recombination. Moreover, Wapl repression by PAX5 controls chromatin architecture at a global scale in pro-B cells. This provides a mechanism for previous reports of a global role for PAX5 in progenitor B cell genome organization that was not dependent on its canonical role as a transcription factor (64).

Interestingly, v-Abl-transformed pro-B cell lines have increased transcription of Wapl (61). Degradation of Wapl in these cell lines restores locus contraction and utilization of distal V_H_ genes in V_H_-DJ_H_ recombination. Taken together, PAX5 repression of Wapl facilitates RAG scanning across the entire V_H_ domain, allowing loops much larger than the average TAD. Lower levels of WAPL enable RAG scanning over longer distances due to increased cohesin residency time on DNA, which, at the Igh locus, results in increased locus contraction and facilitates recombination to distal V_H_ genes. Cohesin and CTCF also play key roles in TCR recombination (65), suggesting that loop extrusion may play a wider role in AgR recombination. However it is unclear whether control of loop extrusion through Wapl regulation can be extended to other AgR loci. Indeed, Wapl expression remains high during T cell development, including during TCRβ and TCRα rearrangement (53), thus different mechanisms may be at play to modulate cohesin dynamics. It will be interesting to see if other lymphocyte specific transcription factors regulate the loop extrusion machinery.

## A Different Paradigm? Live CELL IMAGING and Polymer Modeling Suggests That Phase Separation Contributes to Regulation of Recombination

Phase separation describes a concept that a mixture of components can separate based on their physical and chemical properties into two or more distinct phases. It has been proposed that phase separation, driven by cooperative interactions between chromatin associated proteins, underlies A and B compartmental segregation of chromosomal regions (66, 67). Polymer simulations have suggested that the interplay of active chromatin loop extrusion and phase separation mechanisms can explain chromatin organization (66).

Murre and colleagues extended their previous live imaging work, developing a two color labeling strategy to simultaneously track the motion of paired V_H_ and DJ_H_ loci (55). Computer modeling where the Igh locus was in a weak gel state, near the sol-gel phase boundary, best reproduced V_H_-DJ_H_ first encounter times (**Figure 2B**). This phase enables the Igh locus to move flexibly to facilitate rapid and random recombination while providing enough stability to promote ordered D_H_-J_H_ and then V_H_-DJ_H_ recombination. The authors propose that phase separation acts cooperatively with loop extrusion, employing local chromatin based cross-linking to stabilise the loops. However, this model of restrained motion is rather at odds with earlier studies from the lab which are indicative of rapid, flexible motion (36).

## Conclusions and Future Perspectives

In recent years, enormous progress has been made in three key areas: description of Igh locus conformation; understanding the underlying mechanisms that organize the locus; understanding how the RAG recombinase achieves RSS synapsis. Together, these components provide an extremely intricate and dynamic picture of the role of AgR locus conformation in generation of diverse AgR repertoires. In each sphere, divergent models have emerged and future progress will depend on new methods to resolve their differences. First, convergence of the three subdomain and flexible looping models of conformation could be achieved by high resolution capture Hi-C of the entire Igh locus. Second, of the two rather different mechanisms proposed to organize the locus, loop-extrusion, orchestrated by cohesin, CTCF and Wapl, is supported by numerous genome-wide studies. Phase separation is less established and reflects pioneering work. While the convergence of these two models is not on the immediate horizon, it is conceivable, based on polymer modeling studies that have shown the interplay of these two mechanisms genome-wide (65), that loop extrusion and phase separation act cooperatively to co-orchestrate AgR folding and recombination. Here, we envisage that developments in polymer modeling approaches may resolve the differences between these models. Igh locus conformation per se, and its underlying mechanisms are RAG-independent, and serve to prepare the locus for recombination events. The third piece of the puzzle is RAG targeting. Contrasting models suggest that the RAG complex avails of loop extrusion to scan for convergent RSSs, or brings nearby convergent RSSs together by diffusion. Neither model yet fully explains recombination to distal V_H_ genes. Here too, cooperation between two mechanisms may be at play. Gene-targeting models addressing both may reveal how this may occur.

## Author Contributions

CR and AC planned the content of the review and wrote the abstract. CR and OM drafted the manuscript, AC edited the manuscript, CR prepared the figures, CR, OM and AC finalized the manuscript. All authors contributed to the article and approved the submitted version.

## Funding

CR is supported by a PhD studentship (1947339) from the Medical Research Council, UK. OM was supported by a PhD studentship (1426107) from the Medical Research Council, UK. Work in AEC's laboratory is supported by grants from the Biotechnology and Biological Scientific Research Council (BBS/E/B/000C0404, BBS/E/B/000C0405, BBS/E/B/000C0427, BBS/E/B/000C0428). The Babraham Institute provides funds, throough the BBSRC, for open access publication fees.

## Conflict of Interest

The authors declare that the research was conducted in the absence of any commercial or financial relationships that could be construed as a potential conflict of interest.
